# Frontline treatment patterns and attrition rates by subsequent lines of therapy in patients with newly diagnosed multiple myeloma

**DOI:** 10.1186/s12885-020-07503-y

**Published:** 2020-11-10

**Authors:** Rafael Fonseca, Saad Z. Usmani, Maneesha Mehra, Mary Slavcev, Jianming He, Sarah Cote, Annette Lam, Jon Ukropec, Eric M. Maiese, Sandhya Nair, Ravi Potluri, Peter M. Voorhees

**Affiliations:** 1 Division of Hematology and Oncology, Mayo Clinic, 13400 E. Shea Blvd., MCCRB 3-001, Phoenix, AZ 85259 USA; 2 Levine Cancer Institute/Atrium Health, Charlotte, NC USA; 3 Janssen Global Services, LLC, Raritan, NJ USA; 4 Janssen Global Medical Affairs, Horsham, PA USA; 5 Janssen Scientific Affairs, Horsham, PA USA; 6 Janssen Pharmaceutica NV, Beerse, Belgium; 7 SmartAnalyst Inc., New York, NY USA

**Keywords:** Attrition rates, Autologous stem cell transplant, Bortezomib, Dexamethasone, Lenalidomide, Line of therapy, Newly diagnosed multiple myeloma, Treatment duration

## Abstract

**Background:**

For patients with multiple myeloma (MM), each additional line of therapy (LOT) is associated with lower response rates, shorter treatment duration and treatment-free intervals, and increased rates of toxicities and comorbidities. Here, we examine frontline treatment patterns, and attrition rates by LOT among newly diagnosed MM (NDMM) patients in the United States who were eligible or ineligible for autologous stem cell transplant (ASCT).

**Methods:**

Data were identified from three US patient-level databases collectively covering the period January 2000 to September 2018. Patients had an index diagnosis of MM on or after January 1, 2007, medical and prescription insurance coverage at diagnosis, a 1-year look-back period prior to the index diagnosis, no prior malignancies in the 1-year period before index diagnosis, and had received ≥1 LOT.

**Results:**

Among patients who did not receive ASCT (non-transplant; *n* = 22,062), 12,557 (57%) received only 1 LOT and 9505 (43%) received > 1 LOT. Patients receiving only 1 LOT were significantly older, had higher mean Charlson Comorbidity Index (CCI) scores, and higher incidences of comorbidities. Among the 2763 patients receiving ASCT, 2184 received > 1 LOT, and 579 (21%) received only 1 LOT (ie, ASCT was the last treatment). 1682 (61%) patients received induction therapy as frontline treatment, of whom 187 (11%) also received consolidation therapy. The latter group was younger than those who received only induction therapy, had lower mean CCI scores, and comparable or lower incidences of selected comorbidities. The most common frontline therapy for non-transplant and transplant-eligible patients was bortezomib/dexamethasone and bortezomib/lenalidomide/dexamethasone, respectively. Attrition rates across all LOTs were high for non-transplant patients (range, 43–57%) and transplant patients (range, 21–37%). Treatment duration decreased by LOT for non-transplant patients and was consistent across LOTs for transplant patients.

**Conclusions:**

In this analysis, a substantial proportion of patients with NDMM who received frontline therapy did not appear to receive a subsequent LOT. These high attrition rates underscore the need to use the most optimal treatment regimens upfront rather than reserving them for later LOTs in which the clinical benefit may decrease.

## Background

Multiple myeloma (MM) treatment options have improved in recent years, but MM remains an incurable disease with an estimated 5-year survival rate of 52% in the United States [1]. Frontline and subsequent treatment options are selected based on a patient’s age, frailty, comorbidity status, intolerance, resistance, and/or exposure to previous therapy, and disease biology [2, 3]. If a patient is eligible, autologous stem cell transplant (ASCT) is recommended [2, 3]. Although patients who receive a stem cell transplant and maintenance therapy have superior outcomes compared with those who are transplant ineligible [4, 5], both groups of patients frequently experience disease relapse and require subsequent lines of therapy (LOTs).

Clinical trials evaluate the efficacy and safety of a therapy at a given disease stage and can be highly selective for a patient population (ie, by restricting enrollment to patients with good performance status). Although clinical trials provide valuable information for specific therapeutic regimens, there is limited information on the factors guiding treatment patterns and patient outcomes in a real-world setting.

Available real-world data suggest that most patients diagnosed with MM receive frontline therapy (64–95%); however, attrition rates increase with each successive LOT, with an estimated 32–61% and 14–38% of diagnosed patients receiving second- and third-line therapy, respectively [6–8]. The depth of response, time to progression, and duration of treatment decrease with each successive LOT, while the incidences of toxicity increase [6]. Moreover, patient-reported health-related quality of life significantly decreases with each LOT [9]. Together, these data strongly support the use of the most effective therapy upfront.

The impact of newer agents on observed attrition rates has not yet been evaluated. This study utilized the most recent patient-level data available from three databases in the United States, capturing the years 2000 to 2018. Additionally, it characterized and compared newly diagnosed MM (NDMM) patients who did receive ASCT with those who did not by the number of subsequent LOTs received.

## Methods

### Data sources

Patients with NDMM were identified from 3 US databases: the OPTUM™ Commercial Claims database from January 2000–September 2018, the OPTUM™ Electronic Medical Records (EMR) database from January 2000–September 2018, and the Surveillance, Epidemiology, and End Results (SEER)-Medicare Linked database from January 2007–December 2016. The small number of patients who overlapped between the 2 OPTUM™ databases were excluded; Medicare Advantage patients were excluded from the SEER-Medicare database. Patient-level data were included in the assessment if the patient had their first MM diagnosis (defined as their index diagnosis) on or after January 1, 2007, known gender, medical and prescription insurance coverage in place at diagnosis, no previous malignancies in the 1-year period prior to index diagnosis, a 1-year look-back period prior to index diagnosis, and received ≥1 LOT. Non-transplant patients did not receive ASCT at any time during follow-up. For the SEER-Medicare and OPTUM™ commercial claims databases, insurance eligibility information was available, and a patient’s follow-up was considered to end when they no longer had continuous eligibility. For the OPTUM™ EMR data, follow-up ended on the last date of observed activity for the patient.

### Measures

An LOT was identified based on an initial administration of ≥1 anti-myeloma agent that continued until ≥1 agent was discontinued for ≥60 days or until a new agent was administered. The additional rules that were applied to create these LOTs are included in Additional file 1. For transplant patients, consolidation therapy was defined as ongoing treatment (occurring within 4 months of ASCT) for ≥1 month after ASCT, and was the same regimen that was used for induction therapy or was a new regimen that included the induction therapy. Attrition rate was defined as the ratio of patients who did not have record of a subsequent MM LOT because of death or loss to follow-up (ie, no subsequent treatment in follow-up) in the database for any reason. Comorbidities that were considered clinically relevant, the Charlson Comorbidity Index score [10], age at index diagnosis, frontline treatment regimens (eg, bortezomib/dexamethasone [Vd]), and attrition rates at each subsequent LOT up to LOT5 were characterized. Comorbid conditions were evaluated with the use of CCI score. Comorbidities were stratified by age in the non-transplant and transplant patients. Duration of treatment (defined as the number of months from the start to the end of a given LOT) was evaluated at each subsequent LOT.

### Statistical analysis

For demographics and pre-existing comorbidities in non-transplant and transplant patients with NDMM by LOT, Mantel-Haenszel chi-squared tests for categorical variables and t-tests for continuous variables were conducted. Logistic regression analyses were conducted to characterize and compare patients who did not receive a subsequent LOT after LOT1 with those who received LOT2 and beyond. To account for censoring, a sensitivity analysis was conducted in which the occurrence of a subsequent treatment for patients who had follow-up data available for at least 6+, 12+, and 24+ months was evaluated. For this analysis, cardiovascular disorders (cardiac arrythmia, congestive heart failure, complicated hypertension, and valvular disease) were consolidated into a single variable to reduce the number of covariates in the model. Attrition rates were reported for patients who received transplant and up to five LOTs.

## Results

### Patients and baseline characteristics

This analysis included patient-level data from patients with NDMM who did not receive ASCT (non-transplant, *n* = 22,062) and who received ASCT (transplant, *n* = 2763). Patient demographics and pre-existing comorbidities by LOT are shown in Table 1. The median (Q1–Q3) age was 72.0 (65.0–79.0) years for non-transplant patients and 65.0 (57.0–69.0) years for transplant patients. Non-transplant patients had a higher mean (SD) baseline score compared with transplant patients (1.5 [1.8] vs. 1.2 [1.7]). Non-transplant patients who received only 1 LOT were significantly older than those who received > 1 LOT (median [Q1–Q3] age, 73.0 [65.0–80.0] years vs. 72.0 [65.0–78.0] years; *p* < 0.0001), and had higher mean CCI scores and higher incidences of all comorbidities except simple hypertension (Table 1). Similarly, transplant patients who received only 1 LOT were older than those who received > 1 LOT (median [Q1–Q3] age, 65.0 [57.0–69.0] years vs. 64.0 [57.0–69.0] years) and had higher mean CCI scores and higher incidences of all comorbidities except congestive heart failure (Table 1).

**Table 1:** Demographics and pre-existing comorbidities in non-transplant and transplant patients with NDMM by LOT

	Non-transplant				Transplant							
Characteristic	Non-transplant overall *N* = 22,062	By use of LOT			Transplant overall *N* = 2763	By use of LOT			Frontline induction therapy	Frontline induction therapy by use of consolidation therapy^a^		Frontline induction therapy
		> 1 LOT *n* = 9505	Only 1 LOT *n* = 12,557	*P*-value^b^		> 1 LOT *n* = 2184	Only 1 LOT *n* = 579	*P*-value^b^	Yes *n* = 1682	Yes *n* = 187	No *n* = 1495	No *n* = 1081
Age at MM diagnosis, years												
Mean (SD)	71.1 (10.4)	70.4 (10.1)	71.6 (10.6)	< 0.0001	62.3 (8.7)	62.3 (8.6)	62.4 (9.0)	0.6397	62.9 (8.6)	61.0 (9.1)	63.1 (8.5)	61.4 (8.8)
Median (Q1–Q3)	72.0 (65.0–79.0)	72.0 (65.0–78.0)	73.0 (65.0–80.0)	< 0.0001	65.0 (57.0–69.0)	64.0 (57.0–69.0)	65.0 (57.0–69.0)	0.3294	65.0 (58.0–69.0)	62.0 (55.0–68.0)	65.0 (58.0–69.0)	63.0 (56.0–68.0)
Min, max	20, 100	23, 95	20, 100		27, 82	27, 82	31, 80		27, 82	37, 80	27, 82	28, 80
Age categories, *n* (%)				< 0.0001				0.7143				
< 65	5197 (23.6)	2304 (24.2)	2893 (23.0)		1378 (49.9)	1094 (50.1)	284 (49.1)		781 (46.4)	NR	NR	597 (55.2)
65–74	7741 (35.1)	3581 (37.7)	4160 (33.1)		1298 (47.0)	1019 (46.7)	279 (48.2)		840 (49.9)	74 (40)	766 (51)	458 (42.4)
75–84	7536 (34.2)	3174 (33.4)	4362 (34.7)		87 (3.1)	71 (3.3)	16 (2.8)		61 (3.6)	NR	NR	26 (2,4)
≥ 85	1588 (7.2)	446 (4.7)	1142 (9.1)		–	–	–		–	–	–	–
Male, *n* (%)	11,097 (50.3)	4731 (49.8)	6366 (50.7)	0.1745	1556 (56.3)	1249 (57.2)	307 (53.0)	0.0723	927 (55.1)	110 (59)	817 (55)	629 (58.2)
Race, *n* (%)^c^				< 0.0001				< 0.0001				
White	13,176 (59.7)	5847 (61.5)	7329 (58.4)		1263 (45.7)	1069 (48.9)	194 (33.5)		709 (42.2)	130 (70)	579 (39)	554 (51.2)
Black	2901 (13.1)	1269 (13.4)	1632 (13.0)		252 (9.1)	203 (9.3)	49 (8.5)		128 (7.6)	25 (13)	103 (7)	124 (11.5)
Asian	428 (1.9)	205 (2.2)	223 (1.8)		26 (0.9)	NR	NR		NR	NR	NR	NR
Hispanic	225 (1.0)	108 (1.1)	117 (0.9)		NR	NR	NR		NR	NR	NR	NR
Unknown/other	5332 (24.2)	2076 (21.8)	3256 (25.9)		1207 (43.7)	880 (40.3)	327 (56.5)		821 (48.8)	29 (16)	792 (53)	386 (35.7)
Charlson Comorbidity Index												
Mean (SD)	1.5 (1.8)	1.4 (1.7)	1.5 (1.8)	0.0065	1.2 (1.5)	1.1 (1.4)	1.3 (1.7)	0.0306	1.2 (1.5)	0.8 (1.5)	1.3 (1.5)	1.1 (1.4)
Median (Q1–Q3)	1.0 (0–2.0)	1.0 (0–2.0)	1.0 (0–2.0)	0.5335	1.0 (0–2.0)	1.0 (0–2.0)	1.0 (0–2.0)	0.2183	1.0 (0–2.0)	0.0 (0–1.0)	1.0 (0–2.0)	0.0 (0–2.0)
Min, max	0, 13	0, 12	0, 13		0, 11	0, 10	0, 11		0, 11	0, 8	0, 11	0, 10
Comorbidities during 180 days prior to start of first LOT, *n* (%)												
Any comorbidity below	14,539 (65.9)	6164 (64.9)	8375 (66.7)	0.0042	1638 (59.3)	1261 (57.7)	377 (65.1)	0.0013	1051 (62.5)	82 (44)	969 (65)	587 (54.3)
Cardiac arrhythmia	4327 (19.6)	1627 (17.1)	2700 (21.5)	< 0.0001	294 (10.6)	232 (10.6)	62 (10.7)	0.9528	182 (10.8)	16 (9)	166 (11)	112 (10.4)
Congestive heart failure	2984 (13.5)	1008 (10.6)	1976 (15.7)	< 0.0001	117 (4.2)	95 (4.3)	22 (3.8)	0.5589	63 (3.7)	NR	NR	54 (5.0)
Hypertension, complicated	3718 (16.9)	1382 (14.5)	2336 (18.6)	< 0.0001	260 (9.4)	177 (8.1)	83 (14.3)	< 0.0001	165 (9.8)	NR	NR	95 (8.8)
Hypertension, simple	11,698 (53.0)	5059 (53.2)	6639 (52.9)	0.6022	1316 (47.6)	1016 (46.5)	300 (51.8)	0.0234	847 (50.4)	62 (33)	785 (53)	469 (43.4)
Hepatic disease	972 (4.4)	388 (4.1)	584 (4.7)	0.0415	151 (5.5)	102 (4.7)	49 (8.5)	0.0004	104 (6.2)	NR	NR	47 (4.3)
Pulmonary circulation disorders	1027 (4.7)	348 (3.7)	679 (5.4)	< 0.0001	56 (2.0)	39 (1.8)	17 (2.9)	0.0807	32 (1.9)	NR	NR	24 (2.2)
Renal impairment	5317 (24.1)	2083 (21.9)	3234 (25.8)	< 0.0001	471 (17.0)	352 (16.1)	119 (20.6)	0.0116	298 (17.7)	23 (12)	275 (18)	173 (16.0)
Valvular disease	2258 (10.2)	887 (9.3)	1371 (10.9)	0.0001	158 (5.7)	119 (5.4)	39 (6.7)	0.2357	92 (5.5)	NR	NR	66 (6.1)

*LOT* Line of therapy, *MM* Multiple myeloma, *NDMM* Newly diagnosed multiple myeloma, *NR* Not reported, *Q* Quartile, *SD* Standard deviation

Values of 1–10 have not been reported to conform to the Centers for Medicare and Medicaid Services cell size suppression policy; Similarly, data have not been reported for categorical variables and by subgroup to avoid making it possible to calculate the values of the NR cells

^a^Consolidation therapy was evaluated among patients with NDMM who received frontline induction therapy, and was required to have been ongoing for ≥1 month after ASCT

^b^*P*-value vs. > 1 LOT; *P-*values were computed using chi-squared tests for categorical variables and *t*-tests for continuous variables

^c^Race data were not available in the OPTUM™ Commercial Claims database for patients who received transplant

Of the 2763 transplant-eligible patients, 1682 (60.9%) received a transplant with frontline induction therapy. Of the transplant patients who received frontline induction therapy, 187 (11.1%) also received consolidation therapy. Patients who received consolidation therapy were younger compared with those who did not receive consolidation (median [Q1–Q3] age, 62.0 [55.0–68.0] years vs. 65.0 [58.0–69.0] years), had lower mean CCI and comparable or lower incidences of selected comorbidities (Table 1).

More non-transplant patients had comorbidities during the 180 days prior to their MM diagnosis compared with transplant patients (cardiac arrhythmia, 19.6% vs. 10.6%; congestive heart failure, 13.5% vs. 4.2%; hypertension [complicated], 16.9% vs. 9.4%; and renal impairment, 24.1% vs. 17.0%) (Table 2). When stratified by age, non-transplant patients generally presented with more comorbidities than transplant patients within the same age group. However, the differences were less pronounced in subgroups of patients who were 65–74 and 75–84 years of age, with a higher incidence of some pre-existing comorbidities observed among transplant patients (Table 2).

**Table 2:** Pre-existing comorbidities in non-transplant and transplant patients with NDMM stratified by age

	Overall		< 65 years		65–74 years		75–84 years		85+ years	
	Non-transplant (*N* = 22,062)	Transplant (*N* = 2763)	Non-transplant (*n* = 5197)	Transplant (*n* = 1378)	Non-transplant (*n* = 7741)	Transplant (*n* = 1298)	Non-transplant (*n* = 7536)	Transplant (*n* = 87)	Non-transplant (*n* = 1588)	Transplant (*n* = 0)
Charlson Comorbidity Index during 180 days prior to index MM diagnosis										
Mean (SD)	1.5 (1.8)	1.2 (1.5)	0.9 (1.6)	0.7 (1.3)	1.5 (1.8)	1.6 (1.6)	1.6 (1.8)	1.5 (1.3)	2.1 (1.9)	–
Median (Q1–Q3)	1.0 (0–2.0)	1.0 (0–2.0)	0 (0–1.0)	0 (0–1.0)	1.0 (0–2.0)	2.0 (0–2.0)	1.0 (0–2.0)	2.0 (0–2.0)	2.0 (0–3.0)	–
Min, max	0, 13	0, 11	0, 13	0, 9	0, 12	0, 11	0, 12	0, 5	0, 10	–
Comorbidities during 180 days prior to index MM diagnosis, *n* (%)										
Any comorbidity below	14,539 (65.9)	1638 (59.3)	2556 (49.2)	746 (54.1)	5186 (67.0)	826 (63.6)	5476 (72.7)	66 (75.9)	1321 (83.2)	–
Cardiac arrhythmia	4327 (19.6)	294 (10.6)	529 (10.2)	139 (10.1)	1396 (18.0)	143 (11.0)	1854 (24.6)	12 (13.8)	548 (34.5)	–
Congestive heart failure	2984 (13.5)	117 (4.2)	493 (9.5)	NR	956 (12.3)	59 (4.5)	1179 (15.6)	NR	356 (22.4)	–
Hypertension, complicated	3718 (16.9)	260 (9.4)	603 (11.6)	NR	1274 (16.5)	143 (11.0)	1435 (19.0)	NR	406 (25.6)	–
Hypertension, simple	11,698 (53.0)	1316 (47.6)	1871 (36.0)	569 (41.3)	4215 (54.5)	692 (53.3)	4490 (59.6)	55 (63.2)	1122 (70.7)	–
Hepatic disease	972 (4.4)	151 (5.5)	269 (5.2)	NR	392 (5.1)	71 (5.5)	241 (3.2)	NR	70 (4.4)	–
Pulmonary circulation disorders	1027 (4.7)	56 (2.0)	172 (3.3)	NR	357 (4.6)	23 (1.8)	388 (5.1)	NR	110 (6.9)	–
Renal impairment	5317 (24.1)	471 (17.0)	932 (17.9)	230 (16.7)	1829 (23.6)	222 (17.1)	2028 (26.9)	19 (21.8)	528 (33.2)	–
Valvular disease	2258 (10.2)	158 (5.7)	282 (5.4)	NR	744 (9.6)	90 (6.9)	966 (12.8)	NR	266 (16.8)	–

*MM* Multiple myeloma, *NDMM* Newly diagnosed multiple myeloma, *NR* Not reported, *Q* Quartile, *SD* Standard deviation

Values of 1–10 have not been reported in any cell in this table to conform to the Centers for Medicare and Medicaid Services cell size suppression policy; Similarly, values have not been reported for categorical variables and by subgroup to avoid making it possible to calculate the values of the NR cells

For non-transplant patients, logistic regression analysis showed that several baseline characteristics and comorbidities were associated with receiving further LOT. In the overall population, patients with older age, cardiovascular disorders, pulmonary circulation disorders, and renal impairment were less likely to receive further LOT. For the sensitivity analyses, in which patients who had 6+, 12+, and 24+ months of follow-up data were restricted, older age and cardiovascular disorders were associated with higher attrition rates (Table 3). Among transplant-eligible patients, younger age (< 65 years) was a predictor of subsequent treatment, with patients significantly more likely to receive subsequent therapy at the 12+ and 24+ month follow-up. Liver disease was associated with higher attrition rates for the overall population and for patients with 6+, 12+, and 24+ months of follow-up data (Table 3).

**Table 3:** Predictors of subsequent treatment for non-transplant and transplant patients with NDMM

	Non-transplant patients							
	Overall (*N* = 22,062)		6+ months follow-up^a^ (*n* = 14,866)		12+ months follow-up^a^ *(n* = 11,619)		24+ months follow-up^a^ (*n* = 7233)	
Predictors of subsequent treatment (receiving LOT2+ vs. only LOT1)	Odds ratio^b^ (95% CI)	*P*-value^b^	Odds ratio^b^ (95% CI)	*P*-value^b^	Odds ratio^b^ (95% CI)	*P*-value^b^	Odds ratio^b^ (95% CI)	*P*-value^b^
Age at MM diagnosis, years								
< 65	0.90 (0.84–0.97)	0.0035	1.01 (0.92–1.11)	0.7975	1.09 (0.98–1.22)	0.1154	1.08 (0.94–1.25)	0.2889
65–74	Reference		Reference		Reference		Reference	
75–84	0.86 (0.80–0.91)	< 0.0001	0.84 (0.77–0.90)	< 0.0001	0.86 (0.78–0.94)	0.0010	0.91 (0.80–1.03)	0.1173
≥ 85	0.47 (0.42–0.53)	< 0.0001	0.45 (0.40–0.52)	< 0.0001	0.44 (0.38–0.52)	< 0.0001	0.55 (0.44–0.69)	< 0.0001
Sex, male	0.96 (0.91–1.01)	0.1494	1.03 (0.96–1.10)	0.3843	1.08 (1.00–1.17)	0.0509	1.10 (0.99–1.22)	0.0871
Comorbidities during 180 days prior to index MM diagnosis								
Cardiovascular disorders^c^	0.84 (0.79–0.90)	< 0.0001	0.84 (0.78–0.91)	< 0.0001	0.83 (0.76–0.91)	< 0.0001	0.88 (0.78–1.00)	0.0479
Liver disease	0.91 (0.80–1.04)	0.1573	0.88 (0.75–1.03)	0.1195	0.88 (0.72–1.06)	0.1784	0.73 (0.57–0.94)	0.0149
Pulmonary circulation disorders	0.75 (0.66–0.86)	< 0.0001	0.76 (0.64–0.89)	0.0009	0.75 (0.62–0.91)	0.0030	0.79 (0.60–1.04)	0.0924
Renal impairment	0.91 (0.85–0.98)	0.0112	0.91 (0.83–0.99)	0.0225	0.97 (0.88–1.07)	0.5209	0.94 (0.82–1.07)	0.3363
	**Transplant patients**							
	**Overall (*N*** **= 2763)**		**6+ months follow-up**^**a**^ **(*n*** **= 2595)**		**12+ months follow-up**^**a**^ **(*n*** **= 2245)**		**24+ months follow-up**^**a**^ **(*n*** **= 1535)**	
**Predictors of subsequent treatment (receiving LOT2+ vs. only LOT1)**	**Odds ratio**^**b**^ **(95% CI)**	***P*-value**^**b**^	**Odds ratio**^**b**^ **(95% CI)**	***P*-value**^**b**^	**Odds ratio**^**b**^ **(95% CI)**	***P*-value**^**b**^	**Odds ratio**^**b**^ **(95% CI)**	***P*-value**^**b**^
Age at MM diagnosis, years								
< 65	1.05 (0.87–1.27)	0.5989	1.13 (0.92–1.38)	0.2421	1.30 (1.03–1.65)	0.0288	1.40 (1.02–1.91)	0.0380
65–74	Reference		Reference		Reference		Reference	
75–84	1.21 (0.69–2.12)	0.5059	1.00 (0.57–1.77)	0.9953	1.11 (0.55–2.23)	0.7656	0.75 (0.34–1.67)	0.4855
≥ 85	–	–	–	–	–	–	–	–
Sex, male	1.20 (1.00–1.44)	0.0543	1.18 (0.97–1.45)	0.1029	1.18 (0.93–1.49)	0.1650	1.27 (0.94–1.73)	0.1218
Comorbidities during 180 days prior to index MM diagnosis								
Cardiovascular disorders^c^	0.85 (0.68–1.08)	0.1790	0.86 (0.67–1.11)	0.2423	0.88 (0.66–1.17)	0.3664	0.99 (0.68–1.45)	0.9528
Liver disease	0.56 (0.39–0.81)	0.0017	0.49 (0.34–0.70)	0.0001	0.48 (0.31–0.73)	0.0005	0.41 (0.23–0.72)	0.0020
Pulmonary circulation disorders	0.70 (0.39–1.27)	0.2466	0.60 (0.33–1.09)	0.0926	0.51 (0.26–1.01)	0.0520	0.36 (0.14–0.90)	0.0281
Renal impairment	0.79 (0.62–1.02)	0.0668	0.71 (0.54–0.92)	0.0099	0.74 (0.55–1.01)	0.0601	0.58 (0.40–0.86)	0.0059

*CI* Confidence interval, *LOT* Line of therapy, *MM* Multiple myeloma

^a^Months of follow-up were calculated from the start of the first line of treatment until either the end of insurance eligibility (for SEER and OPTUM™ Commercial)

^b^Odds ratios and *p*-values were computed using a logistic regression

^c^Cardiovascular disorders include cardiac arrythmia, congestive heart failure, complicated hypertension, and valvular disease

### Frontline treatment regimens

**Fig. 1:**
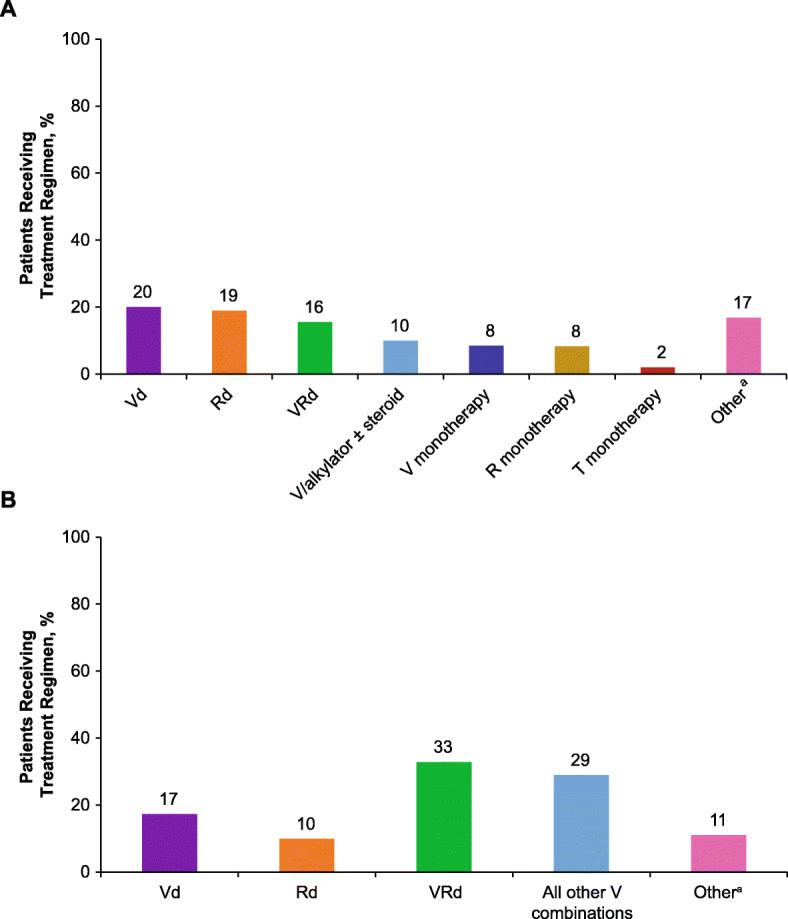
Frequently used frontline treatment regimens for patients with NDMM (**a**) who did not receive stem cell transplant (non-transplant) and (**b**) who received stem cell transplant (transplant). *NDMM* Newly diagnosed multiple myeloma, *Vd* Bortezomib/dexamethasone, *Rd* Lenalidomide/dexamethasone, *VRd* Bortezomib/lenalidomide/dexamethasone, *V/alkylator* Bortezomib/alkylating agent, *V* Bortezomib, *R* Lenalidomide, *T* Thalidomide. ^a^Other regimens include other combinations of novel agents, such as Td and VR, as well as treatment with cyclophosphamide-containing regimens

For non-transplant patients, frontline treatment regimens varied, with Vd (20%) and lenalidomide/dexamethasone (Rd; 19%) being the most common (Fig. 1a). The most common induction treatment regimens for transplant patients were bortezomib/lenalidomide/dexamethasone (VRd; 33%) and other bortezomib-containing regimens (29%) (Fig. 1b). Among patients who received consolidation therapy (ongoing for ≥1 month after ASCT, and the same regimen as used for induction, or a new regimen which included the induction therapy), the majority received either VRd (41%) or other bortezomib-containing regimens (38%).

### Attrition by LOT

Attrition rates across all LOTs were high for non-transplant patients (range, 43–57%) and transplant patients (range, 21–37%); rates of attrition were higher for the non-transplant patients (Table 4). After frontline therapy, 43% of non-transplant patients and 79% of transplant patients received a second LOT, and of these, only 55% and 69% went on to receiving a third LOT, respectively. Only 8% of total non-transplant patients and 22% of total transplant patients received a fifth LOT. The incidence of death was higher at each LOT for non-transplant (19–22%) vs. transplant patients (3–12%), with comparable treatment duration (Table 4).

**Table 4:** Attrition rates by LOT

LOT	Frequency, *N*	Attrition, %	Deaths, *n* (%)	No subsequent treatment in follow-up, *n* (%)	Subsequent treatment, *n* (%)	Mean ± SD treatment duration, months (median)
Non-transplant						
1	22,062	–	2841 (12.9)	9716 (44.0)	9505 (43.1)	6.9 ± 9.6 (3.6)
2	9505	56.9	1155 (12.2)	3168 (33.3)	5182 (54.5)	7.5 ± 9.5 (4.1)
3	5182	45.5	636 (12.3)	1575 (30.3)	2971 (57.3)	6.5 ± 8.0 (3.7)
4	2971	42.7	364 (12.3)	901 (30.3)	1706 (57.4)	5.7 ± 6.6 (3.4)
5	1706	42.6	209 (12.3)	508 (29.8)	989 (58.0)	5.5 ± 6.4 (3.2)
Transplant						
1	2763	–	36 (1.3)	543 (19.6)	2184 (79.0)	6.3 ± 8.0 (4.2)
2	2184	21.0	60 (2.7)	613 (28.1)	1511 (69.2)	6.1 ± 9.2 (2.7)
3	1511	30.8	63 (4.2)	494 (32.7)	954 (63.1)	7.4 ± 9.8 (3.6)
4	954	36.9	60 (6.3)	276 (28.9)	618 (64.8)	6.6 ± 9.4 (3.4)
5	618	35.2	49 (7.9)	180 (29.1)	389 (62.9)	5.6 ± 6.2 (3.3)

*LOT* Line of therapy, *SD* Standard deviation

**Fig. 2:**
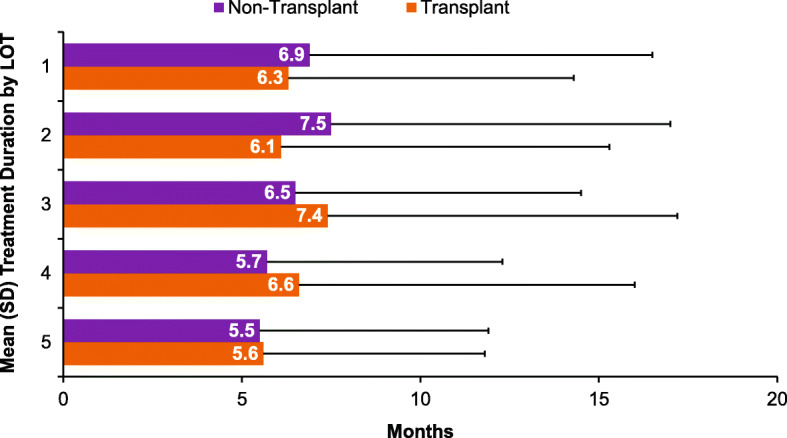
Mean treatment duration by LOT in patients with NDMM who did not receive stem cell transplant (non-transplant) and those who did receive stem cell transplant (transplant). *LOT* Line of therapy, *SD* Standard deviation

For non-transplant patients, the duration of treatment decreased with each LOT, except for the duration of the second LOT (6.9 months for frontline treatment, 7.5 months for LOT2, and 6.5 months for LOT3) (Fig. 2). For patients who received transplant, treatment duration was relatively constant by LOT; with a mean duration of 6.3 months and 6.1 months for the first two LOTs, respectively (Fig. 2).

## Discussion

This retrospective analysis of real-world data from the United States demonstrates that attrition rates were high (up to 50% per LOT) and remained steady from the first through the fifth LOT among non-transplant patients. For transplant patients, attrition rates were generally lower, but increased with each successive LOT, ranging from 21–37%. The difference in attrition for non-transplant and transplant patients is not unexpected, given the different baseline characteristics and comorbidities of these two patient populations. For non-transplant patients, older age at diagnosis, cardiovascular disorders, pulmonary circulation disorders, and renal impairment were associated with higher attrition rates beyond the first LOT. For transplant patients, liver disease was associated with higher attrition rates. With longer follow-up, younger transplant patients were more likely to receive a second LOT, and pulmonary circulation disorders and renal impairment were associated with higher attrition rates.

Among non-transplant patients who received frontline therapy, approximately half received a second LOT and as few as 8% received a fifth LOT. For transplant patients, the proportions in subsequent therapies at each LOT were higher; 79% received a second LOT and 22% received a fifth LOT. In similar analyses of European patients with NDMM, 32–61%, 14–38%, 15%, and 1% received a second, third, fourth, and fifth LOT [6–8, 11]. These estimates included patients with NDMM from multiple countries and, in some instances, did not differentiate between transplant and non-transplant patients. This analysis of the most recent patient-level data (up to 2018) available from three databases in the United States demonstrates a clear difference in attrition rates among transplant-ineligible and transplant-eligible patients. There was a slight increase in patients receiving subsequent LOTs, although it should be noted that the study included only those patients with NDMM who had received at least one LOT rather than all patients diagnosed with MM. Nevertheless, our analysis is consistent with previous real-world studies [6–8, 11] and demonstrates the occurrence of high attrition rates in early lines of MM therapy. Treatment durations in our study were also generally consistent with other real-world analyses [6, 7, 12].

Vd and Rd were the most common standard-of-care (SoC) frontline treatments for patients who did not receive ASCT; the use of Rd as frontline therapy in this setting is consistent with current guidelines [13], although the use of triplets is increasing, and use of Vd is consistent with another real-world analysis reporting that non-transplant patients received Vd as frontline therapy (13.5%) [14]. However, these therapies are no longer optimal. For patients who received ASCT, the most common induction therapies were VRd and other bortezomib-containing regimens. These treatment patterns reflect current guidelines recommending that induction therapy include a minimum of bortezomib and dexamethasone [13]. For patients who received frontline induction therapy plus ASCT, the use of consolidation therapy was limited, seen in only 11% of patients. In our analysis, patients who received consolidation therapy were younger at diagnosis, had lower CCI scores, and had lower rates of cardiac arrhythmia, simple hypertension, pulmonary circulation disorders, and renal impairment. Although this estimate appears low, it is within range of previous real-world analyses (range, 2–22%) [11, 15].

Several limitations should be considered for this retrospective analysis of claims data. Claims data in real-world analyses inherently reflect the individual decisions of physicians as they evaluate and treat patients, which differ from the controlled and standardized methods used in clinical studies. In this real-world dataset, the incidence of pre-existing renal insufficiency was higher in non-transplant than transplant patients, which may or may not be attributable to MM. Furthermore, algorithms were used to establish LOTs, the details of which are included in Additional file 1. This may have led to the overestimation of attrition, as it included patients who were lost to follow-up due to a switch in insurance plans, loss of insurance coverage, or end of the study period. Attrition rates may also have been overstated because the database did not include information on patients’ disease progression; it was therefore not possible to distinguish between patients who did not experience disease progression and did not receive subsequent treatment and those who had disease progression but did not receive subsequent treatment during the study period. It is possible that some of the MM patients who received frontline therapy may have had smoldering MM. Moreover, this study excluded patients with NDMM who opted to forgo therapy. As such, the data herein should be interpreted in the context of patients who received at least one LOT, rather than the entire MM population. Lastly, comparisons within the same LOT between non-transplant and transplant patients, and comparisons of different LOTs within the transplant group, were not adjusted for exposure, and hence should be interpreted with caution.

## Conclusion

High attrition rates for frontline and subsequent LOTs for MM, coupled with known decreases in overall survival, progression-free survival, and response to treatment with each LOT [6, 8, 14, 16] suggest that the most effective treatment is needed early to provide each patient the best opportunity for durable disease control and improved survival. As newer regimens evolve and are adopted into clinical practice as frontline therapies, further analyses of real-world data should be conducted to confirm whether improved outcomes and decreased attrition rates are associated with the earlier use of these superior therapies in the treatment paradigm.

## Supplementary information

**Additional file 1.** Business rules to define line of treatment in multiple myeloma [17–19].

## Data Availability

The data sharing policy of Janssen Pharmaceutical Companies of Johnson & Johnson is available at https://www.janssen.com/clinical-trials/transparency. As noted on this site, requests for access to the study data can be submitted through Yale Open Data Access (YODA) Project site at http://yoda.yale.edu.

## Abbreviations

ASCT: Autologous stem cell transplant
CCI: Charlson Comorbidity Index
LOT: Line of therapy
MM: Multiple myeloma
NDMM: Newly diagnosed multiple myeloma
SOC: Standard of care
Vd: Bortezomib/dexamethasone
VRd: Bortezomib/lenalidomide/dexamethasone
