# Supplementary material for: Frontline treatment patterns and attrition rates by subsequent lines of therapy in patients with newly diagnosed multiple myeloma
Source: BMC Cancer. 2020 Nov 10;20:1087. doi: 10.1186/s12885-020-07503-y (PMC7656738; doi:10.1186/s12885-020-07503-y)
Supplement: Supplementary file 1 — Additional file 1. Business rules to define line of treatment in multiple myeloma [17–19]. [file 12885_2020_7503_MOESM1_ESM.docx]

## Additional file 1

## Business rules to define line of treatment in multiple myeloma.

Previous studies have used similar rules to define the lines of treatment (17-19); a detailed description of these rules is included in this report.

The usage of each core MM drug, steroid and other MM drug will be analyzed individually:

1. Treatment period for a drug would be the period from the first date of use to the last date of use. The last date of use is the earliest date when there is at least a 60-day gap between the day the supply of a claim for a drug is exhausted and the day of the next claim for the same drug. See 1c for imputation rules for assigning the days of supply for a particular drug.
2. A drug used multiple times, except for dexamethasone, with a gap of more than 60 days will have multiple treatment periods (start dates and end dates). If the drug is dexamethasone, a gap of 90 days was considered.
3. For an oral therapy, the end date for each treatment period would be the last Rx date in that treatment period + days supply (days supply is assumed to be 30 days in cases where this data is missing or where days supply is less than 15 days) for all oral therapies, while that for injectable would be the last Rx date plus estimated clinical benefit of 30 days.
4. Once the different treatment periods of each multiple myeloma drug have been identified, these periods are lined up such that the entire treatment duration for a patient is broken up into multiple intervals, each with a distinct regimen (ie, a single drug or a combination of drugs). Each successive interval is deemed to be a different line of treatment for a patient, subject to the below stipulations:
5. If a regimen comprises a core drug(s) along with either a steroid or an “other MM drug,” the regimen will be replaced with only the core drug(s). In other words, the steroid or the other MM drug will be deleted.
6. If a regimen comprises an “other MM drug(s)” along with a steroid, the regimen will be replaced with only the “other MM drug(s).” In other words, the steroid will be deleted from the regimen.
7. Upon completion of these steps, all regimens that are completely identical and are successive (without any blank or other regimens between them) will be merged into one regimen; this regimen will have the minimum start date and maximum end date amongst the successive regimens.
8. If any regimen has a duration of ≤30 days, and if its succeeding regimen is a step-up or step-down regimen, then the 2 regimens should be merged, with the regimen with the higher duration being considered the prevailing regimen. If the succeeding regimen is not a step-up or step-down regimen, then the regimen should be compared with the preceding regimen and the same rule applied. For 2 regimens to be considered step-up or step-down of each other, one of the regimens must be an entire subset of the other regimen. Upon completion of this step, all regimens that are completely identical and successive will be merged into one regimen.
9. If a regimen comprises only an “other MM drug(s)” or only a steroid(s) and the duration is ≤30 days, the regimen will be disregarded and replaced by a blank. Otherwise, the regimen will stand and no change or adjustment will be made.
10. If there is a blank regimen of less than 60 days:
    1. If preceding and succeeding regimens are the same; then the preceding regimen will be deemed to continue until the end date of the regimen succeeding the blank regimen.

(RATIONALE FOR THE ABOVE: During treatment with any regimen, a gap of up to 60 days could exist on account of compliance or adverse events. Therefore, i) if the preceding and succeeding regimens are similar, the 2 periods would be treated as continuous; ii) if preceding and succeeding regimen are not similar then they would be treated as 2different regimens.

1. Sandwich rule - This rule will be applied if either of the following conditions are satisfied:
2. Middle regimen contains dexamethasone. The following conditions need to be met:
   1. There are 3 regimens – regimen1, regimen2, and regimen3, which occur without any gap between them.
   2. AND Regimen1, Regimen3 are core regimens and do not contain dexamethasone and regimen1=regimen3
   3. AND regimen 2 consists of dexamethasone. Regimen2 would be identical to regimen1, regimen3 if dexamethasone were removed.
   4. AND duration of regimen2 is ≤90 days.
3. Middle regimen does not contain dexamethasone. The following conditions need to be met:
   1. There are 3 regimens – regimen1, regimen2, and regimen3, which occur without any gap between them.
   2. AND Regimen1, Regimen3 are core regimens and contain dexamethasone and regimen1=regimen3
   3. AND Regimen2 does not contain dexamethasone. Regimen2 would be identical to regimen1, regimen3 if they did not contain dexamethasone
   4. AND duration of regimen 2 is ≤90 days.

If any of the above rules are satisfied, then regimen 1, regimen2, regimen 3 will be combined as one regimen where regimen 1 and 3 will be the prevailing regimen with start date of regimen 1 and end date of regimen 3.

1. Step-down because of the removal of dexamethasone: If there is a regimen (regimen1) immediately followed (i.e., with no gap) by the same regimen with the removal of dexamethasone only (regimen2), and duration of regimen2 is ≤90 days, then regimen1 and regimen2 will be combined, with regimen1 being the prevailing regimen.
2. Frontline steroids: If the first line of therapy is a steroid, then the regimen will be deleted and the regimens renumbered such that a non-steroid regimen is the first line.
3. All steroid-only regimens will be deleted.
4. If LOT i=LOT (i+1) and they occur within 180 days of each other, the 2 regimens will be combined as one LOT.
5. Additional step-ups and step-downs: The following rules will be applied, in order of hierarchy:
6. If there is at least one PI/IMID/CD38 drug **with DEX** in the regimen followed by the same PI/IMID/CD38 drug **without DEX** in the regimen and they occur within 180 days of each other, the 2 regimens will be combined as PI/IMID/CD38+DEX.

b) If there is at least one PI/IMID/CD38 drug **without DEX** in the regimen followed by the same PI/IMID/CD38 drug **with DEX** in the regimen and they occur within 180 days of each other, the 2 regimens will be combined as PI/IMID/CD38+DEX.

c) If two subsequent regimens occur within 180 days of each other, the 2 regimens will be combined with the initial regimen being the prevailing regimen. This rule will be applied if there is at least one of the following drug classes present in the regimen.

1. If PI + IMID ± DEX is followed by IMID ± DEX, the combined regimen will be PI + IMID ± DEX
2. If CD38 + IMID ± DEX is followed by IMID ± DEX, the combined regimen will be CD38 + IMID ± DEX
3. If CD38 + IMID ± DEX is followed by CD38 ± DEX, the combined regimen will be CD38 + IMID ± DEX
4. If CD38 + PI ± DEX is followed by CD38 ± DEX, the combined regimen will be CD38 + PI ± DEX
5. If CD38 + PI + IMID ± DEX is followed by CD38 + IMID ± DEX, the combined regimen will be CD38 + PI + IMID ± DEX
6. If CD38 + PI + IMID ± DEX is followed by IMID ± DEX, the combined regimen will be CD38 + PI + IMID ± DEX
7. If CD38 + PI + IMID ± DEX is followed by CD38 ± DEX, the combined regimen will be CD38 + PI + IMID ± DEX
8. If CD38 + PI + IMID ± DEX is followed by PI + IMID ± DEX, the combined regimen will be CD38 + PI + IMID ± DEX

d) If two subsequent regimens occur within 180 days of each other and the duration of the initial regimen is ≤180 days, then the 2 regimens will be combined with the initial regimen being the prevailing regimen. This rule will be applied if there is at least one of the following drug classes is present in the regimen.

1. PI + IMID ± DEX is followed by PI ± DEX, the combined regimen will be PI + IMID ± DEX
2. If CD38 + PI ± DEX is followed by PI ± DEX, the combined regimen will be CD38 + PI ± DEX
3. If CD38 + PI + IMID ± DEX is followed by CD38 + PI ± DEX, the combined regimen will be CD38 + PI + IMiD ± DEX
4. If CD38 + PI + IMID ± DEX is followed by PI ± DEX, the combined regimen will be CD38 + PI + IMID ± DEX

e) If second line occurs within 60 days of the initiation of the initial line, the two regimens will be combined as the second line of treatment if the consecutive regimens consist of the following drugs:

1. PI/IMID ± DEX is followed by PI + IMID ± DEX, the combined regimen will be PI + IMID ± DEX
2. PI ± DEX is followed by CD38 + PI ± DEX, the combined regimen will be CD38 + PI ± DEX
3. IMID ± DEX is followed by CD38 + IMID ± DEX, the combined regimen will be CD38 + IMID ± DEX
4. CD38 ± DEX is followed by CD38 + PI ± DEX, the combined regimen will be CD38 + PI ± DEX
5. CD38 ± DEX is followed by CD38 + IMID ± DEX, the combined regimen will be CD38 + IMID ± DEX

**The above rules will be implemented at drug level; ie, same drug should be present in both regimens**.

1. Identical regimens that occur immediately after each other without any gap between them will be combined.
2. Regimens where LOT start date occurs after the death date will be deleted.

**Prednisolone inclusion rule:**

a) Checking for prednisolone overlap: If a regimen includes ≥50% overlap with prednisolone, prednisolone will be tagged on to the regimen.

**List of core drugs**: thalidomide (T), bortezomib (V), lenalidomide (R), melphalan, pomalidomide, carfilzomib, cyclophosphamide, elotuzumab, ixazomib, daratumumab, panobinostat, dexamethasone, doxorubicin

**List of steroids**: prednisone, prednisolone

**List of other MM drugs**: vincristine, etoposide, bendamustine, vorinostat
